# The Effects of Ethanol and Strontium on Growth and Development of Two-Cell Arrested Mouse Embryos

**Published:** 2012-03-20

**Authors:** Mohammad Reza Darabi, Abdolhossein Shiravi, Vida Hojati

**Affiliations:** 1 Department of Anatomy, Arak University of Medical Science, Arak, Iran; 2 Department of Biology, Damghan Branch, Islamic Azad University, Damghan, Iran

**Keywords:** Strontium, Ethanol, Arresting, Cleavage, Development

## Abstract

**Background:**

Arresting at a certain stage of development like the two-cell stage could be one of the causes of infertility. The aim of this study is to evaluate the effects of ethanol and strontium on growth and development of mice embryos arrested at the two-cell stage.

**Materials and Methods:**

In this experimental study, female mice were coupled with a male following superovulation. Positive vaginal plug mice were sacrificed 48 hours after human chorionic gonadotropin (hCG) injection. Two-cell embryos were transferred to M16 medium and divided to four groups. The first control group was incubated without any exposure to low temperatures. Groups 2, 3 and 4 were exposed to 4°C for 24 hours. The second control group was incubated immediately, while the third and fourth groups were exposed to 10 mM strontium for five minutes and 0.1% ethanol for a further five minutes. Growth rate and developmental parameters of embryos were analyzed by one- way ANOVA. The significant difference between the groups was determined by Post Hoc.

**Results:**

The data shows that developmental rate is decreased significantly by 4°C exposure. The mean percentage of degenerated embryo was significantly different between groups but the mean cleavage rate was not significantly different. The mean percent of morula, blastocyst and hatched blastocyst formation were significantly different between groups during a 120 hours study post hCG injection.

**Conclusion:**

The effect of strontium and ethanol on arrested two-cell embryos had no significant effect on the mean percentage of morula, but ethanol treatment significantly increased the percentage of blastocyst and hatched blastocyst formation compared to strontium.

## Introduction

Activators can significantly increase cleavage and developmental rates of parthenogenetic embryos cultured in a medium supplemented with a type of activator. Arrest at the two-cell stage is a topics that is of concern to researchers. In fact arresting of the embryo at two-cell stage occurs in some couples referring for *in vitro* fertilization (IVF) to fertility and infertility centre probably because at the mid-two-cell stage definitive transcription from the zygotic genome occurs in the early embryo (1, 2). The studies on fifty five different strains of mice have shown that there are significant differences in the two-cell stage arresting in different strains (3). Among the factors contributing to this phenomenon, maternal factors play a major role (3, 4). However, the sperm mitochondria, the microtubule-organizing center (MTOC) precursors and the stored cellular components of the sperm do not play a major role in cleavage-stage embryogenesis (5). Thus, the early embryo is almost entirely dependent on the egg for its initial complement of the subcellular organelles and macromolecules that are required for survival prior to the robust activation of the embryonic genome at cleavage-stage development. These maternal components are encoded by maternal effect genes (6,7). Also, studies have shown that the rate of early cleavage depends on reserved mRNA and proteins of oocytes. Arresting causes strong and effective changes in the protein synthesis of the embryo. During this process, maternal signals that cause the cleavage are blocked causing a developmental arrest and embryo degeneration. Studies show that the exposure of oocytes to the media containing activators, results in significantly enhanced cleavage and development rates (8). Strontium has been widely used as an activator of oocytes by mimicking the sperm's function especially in mice. It causes frequent calcium fluctuation in oocytes, releasing cortical granules and finally pronucleus formation, while ethanol continuously causes calcium enhancement. This series of events prevents oocytes from arresting at the first meiotic division stage (9). Embryonic arrest may be another mechanism to prevent further development of certain chromosomally abnormal embryos, and/or embryos that fail to activate their embryonic genome (10). A lot of genes have been identified which are required by the embryo in order to successfully pass all the embryonic developmental stages. The injection of ooplasm from one normal oocyte directly in to an embryo which has arrested at the two-cell stage, removes the blockage and enables the embryo to fully develop (11). Previous studies have indicated that ethanol can activate oocytes and causes parthenogenesis (12- 14). However, ethanol should have a concentration higher than 5% in order to be able to activate oocytes and result in parthenogenesis (12). Ethanol can change the signaling pathway which controls the rate of embryogenesis and can therefore affect the development of preimplantation stage embryos (15). When oocytes are exposed to ethanol, the permeability of the cell membrane increases towards calcium and this increase of intracellular calcium activates the oocyte. Ethanol with contribution to function as secondary messengers, such as calcium, can stimulate the embryos development before implantation. Other oocyte activators exist, such as benzyl alcohol, propanediol and methanol (3). Exposing embryos to low temperatures is one of the causes of developmental arrest. The developmental rate of embryos exposed to low temperature (4ºC) was shown to be significantly decreased (16).

Although strontium chloride is widely used as the reagent inducing oocyte activation in mice, there is controversy over the optimal method for oocyte activation in rats. Different protocols (ethanol, ionomycin, strontium and electrical pulses) have been used for the activation of oocytes obtained from two different strains (SD) and Wistar. The results show that oocytes derived from SD strains have a significantly higher cleavage and blastocyst formation rate compared to those of Wistar strain irrespective of the activation regime used. In both strains, ethanol treatment showed significantly higher developmental ability at cleavage and blastocyst formation compared to other activation protocols. Ethanol treatment is a more optimal protocol for the activation of rat oocytes in different strains (13). Strontium (Sr^2+^) is the most efficient agent for mouse oocyte activation and functions by inducing Ca^2+^ oscillations in both immature and mature oocytes, as well as in early embryos (17). Developmental arrest can be considered as one of the reason for infertility. The aim of this study was to evaluate and compare the effects of ethanol and strontium on the growth and developmental rate of two-cell arrested mouse embryos exposed to low temperature.

## Materials and Methods

In this experimental study, 6 -8 week-old mice (NMRI strain, n=180) were prepared from Razi Vaccine & Serum Research Institute,Iran, and in order to adapt with the new environment, they were kept in the animal facility room of Arak University of Medical Sciences under standard conditions (12 hours light, 12 hours dark, 21 ± 5ºC) for one week (18,19). Female mice were injected intraperitoneally by 10 IU pregnant mare serum gonadotropin (PMSG) for superovulation. After 48 hours they were injected by 10 IU human chorionic gonadotropin (hCG) and coupled with male mice. Positive vaginal plug mice were separated and sacrificed 48 hours after hCG injection by cervical dislocation (20). Two-cell stage embryos were collected in Roswell Park Memorial Institute (RPMI) by flushing of oviduct, transferred to 25 microliter droplets M16 medium (Sigma, M-7292, USA) that were covered with liquid paraffin after 3 time washing. The embryos were divided into four groups: 1 (control 1), 2 (control 2), 3 (experimental 1), and 4 (experimental 2). The embryos of group1 just incubated in 37ºC for 120 hours without any exposure to low temperatures. In order to induce arrest at the two-cell stage, embryos in groups 2, 3 ,and 4 were refrigerated at 4ºC for 24 hours. Group 2 was then cultured at normal conditions while embryos form groups 3 and 4 were exposed to media containing 10 mM of strontium (21) and 0.1% ethanol (22) for five minutes, respectively. The numbers of two-cell embryos in each group were 10-15 and each experiment was repeated 3-5 times. All statistical analyses of this study were analyzed by SPSS software (Version 11.5).Growth rate and developmental parameters of embryos were analyzed by one-way ANOVA. The significant difference between the groups was determined by Post Hoc.

This study was approved by the Ethic Committees of the Institutional Review Board of Islamic Azad University, Damghan Branch.

## Results

The embryos of groups 2, 3 ,and 4 which were exposed to 4ºC for 24 hours, reached blastocyst stage by 18 -24 hours later than embryos in group 1. The results of growth and development of embryos are shown in table 1. The data analysis by one-way ANOVA shows that developmental rate has decreased significantly by exposure to temperatures of 4°C (p = 0.001). The mean percent of degenerated embryo at the two-cell embryos exposed to 4°C increased, and it was significantly different between group 1 with groups 2, 3 and 4 (p=0.004). The mean percentage of cleavage rate did not show any significant differences between groups 3 and 4 (p=0.073). There were also no significant differences in the mean percentage of morula between groups 1-2, 1-3, 1-4, 2-3, 2-4 and 3-4 (p=0.121) while the mean percentage of blastocyst formation was significantly different between group 1 and 2 (p=0.004) and groups 1 and 4 (p=0.009). There were no significant differences between groups 1-3, 2-3, and 3-4 for this parameter. The mean percentage of hatched blastocyst (p=0.002) was significantly different between groups except between groups 2 and 3 (p=0.88).

**Table 1:** The mean percent of degenerated embryos, cleavage, morula, blastocysts and hatched blastocysts in four groups


	Total number	Degenerated embryos%	Cleavage%	Morula%	Blastocysts%	Hatched blastocysts%

**Group 1**	92	11.2 ± 3.2 (10)^b^	91.6 ± 5.4 (84)	79 ± 5 (73)	73.9 ± 8.9(68)^c^	57.3 ± 7.4(53)^c^
**Group 2**	145	32.9 ± 6.3 (49)	69.9 ± 15.4 (10)	65.9 ± 10.3 (97)	48.7 ± 13.2(70)^c^	22.9 ± 10.3(33)
**Group 3**	90	26.7 ± 5.2 (24)	81.7 ± 7.7 (83)	74.5 ± 9.4 (67)	62.8 ± 10.1(56)	20.3 ± 7.2(18)
**Group 4**	59	28 ± 3.1 (17)	82.6 ± 7.4 (49)	75.3 ± 8.9 (44)	54 ± 10.5(32)^b^	40.9 ± 11.5(24)^c^


Value in the parenthesis shows the number of embryo. Values with common superscript are significantly different for that group p≤0.05

## Discussion

The present study has demonstrated that ethanol and strontium can promote the development of two-cell arrested mouse embryos induce by low temperature exposure. The rate of hatched blastocyst formation from two-cell arrested mouse embryos produced by ethanol is significantly higher than strontium; however strontium should potentially support the development of arrested two cells better than ethanol for blastocyst formation. Previous studies show that these two activators have been used extensively for triggering intracellular Calcium fluctuation and subsequently oocyte activation and production of parthenogeneic embryos (13, 14, 23-25). It is possible for the mechanism underlying the embryo activation by ethanol or strontium to be similar and although poorly understood, both of them may have some toxic effects on the growth and development of the embryo. This study shows that the mean percentage of morula derived from arrested two-cell embryos was not significantly different between ethanol and strontium treated groups, in other words they have similar effect on blocked mouse two-cell embryos up to morula stage. The growth and development of *in vitro* produced embryos was slower than *in vivo* possibly due to the deficiency in some maternal growth factors and related nutrients (presenting in fallopian tube and its fluid) *in vitro*, and sensitivity of the early developing embryo to changes in their immediate environment (26, 27). Sakurai et al. have introduced a method of cooling embryos at refrigerated temperatures (short term storage) to keep them in an inactive metabolic state while maintaining their viability (28). These authors showed that the storage of mouse fertilized oocytes at 48ºC for even 48 hours can cause temporary cessation of DNA synthesis while maintaining thier potential to develop to normal blastocysts (28, 29). The results of this study emphasizes on the fact that two-cell mouse embryos exposed to 4ºC undergo developmental arrest and show signs of degeneration and are retarded at the time of blastocyst formation compared to the control group. However, the differences were not significant. It appears that exposure to a temperature of 4ºC, causes a decrease in the cellular metabolism, protein synthesis and metabolic transport system. It can be suggested that under this condition, embryotrophic factors change to embryotoxic factors after a short storage time and increase the sensitivity of embryos to unfavorable compounds of culture (23). With low temperature exposure of embryos, the membrane permeability maybe altered and causes accumulation of some embryotrophic factors in one side of the membrane which can have a toxic effect (30). Interestingly, as amino acids spontaneously break down to produce embryotoxic ammonium ions, the presence of amino acids in the culture medium was also responsible for an inhibition of embryo development (31). In contrast, decreasing number of degenerated embryos in the 4th group compared to the 2nd group is probably caused by the activating effect of ethanol. In concordance with our results, Grabiec et al. have compared the effect of ethanol, magnetic field, calcium ionophore A23187, cycloheximide and a combination of these agents on oocyte activation. The combination of the magnetic field and 10% ethanol for 3 minutes resulted in significantly increased parthenogenetic activation and cleavage rates of rat oocytes more than other treatments (23). Oocytes activated with magnetic field alone gave the lowest activation rate compare with other groups. As a result, magnetic field can be used as a supplementary activating agent, and the combination of ethanol and magnetic field is an effective method for oocyte activation. It is interesting to note that even though Rogers et al. used 7% ethanol for 7 minutes for parthenogenetic activation and development of oocytes, the rate of cleavage, morula and blastocyst formation was the same as the present study (24). Probably supplementary protocols for activating arrested embryos with ethanol could give better results, therefore it would be interesting to conduct a study in order to evaluate a protocol supplemented with magnetic field in the future studies. The study of Zhang et al. was the first report, showing that strontium induced calcium oscillations in early mouse embryos are dedicated by InsP3 receptors, and required phospholipase C activation and a synergistic action of InsP3 (17). Wiebold showed that mouse two-cell stage embryos exposed to 0.1% ethanol had an increased rate of blastocyst formation of up to 86% which is higher than the blastocyts formation rate obatained in our study, this is probably due to low temperature exposure in the present study and decreased metabolic activity (28, 32). Kasai et al. show that *in vitro* mouse embryos exposed to low temperatures above freezing for only a limited period of time can survive and develop to blastocyst stage (33). Krivokharchenko used 2 mM strontium for 2 hours, in order to parthenogenetic activation of rat oocytes in preimplantation stage (25). The mean percentage of cleavage rate was higher than the present study, because of parthenogenesis, arrest and different dose of strontium between these two studies.

## Conclusion

The effect of strontium and ethanol on arrested two-cell embryos has no significant effect on the mean percentage of morula. However unlike strontium, ethanol does significantly increase the mean percentage of blastocyst and hatched blastocyst formation. Finally we propose a study to be conducted in the near future to analyze the effects of other activators on two-cell embryonic development in conjunction with genetical analysis.
